# Pulmonary Findings of [^18^F]FDG PET/CT Images on Asymptomatic COVID-19 Patients

**DOI:** 10.3390/pathogens10070839

**Published:** 2021-07-03

**Authors:** Tzu-Chuan Ho, Chin-Chuan Chang, Hung-Pin Chan, Ying-Fong Huang, Yi-Ming Arthur Chen, Kuo-Pin Chuang, Che-Hsin Lee, Cheng-Hui Yuan, Yu-Zhen Deng, Ming-Hui Yang, Yu-Chang Tyan

**Affiliations:** 1 Department of Medical Imaging and Radiological Sciences, Kaohsiung Medical University, Kaohsiung 807, Taiwan; tzuchuanho@gmail.com (T.-C.H.); huangyf@kmu.edu.tw (Y.-F.H.); jean3092811@gmail.com (Y.-Z.D.); 2 Department of Nuclear Medicine, Kaohsiung Medical University Hospital, Kaohsiung 807, Taiwan; chinuan@gmail.com; 3 School of Medicine, Kaohsiung Medical University, Kaohsiung 807, Taiwan; 4 Neuroscience Research Center, Kaohsiung Medical University, Kaohsiung 807, Taiwan; 5 Department of Electrical Engineering, I-Shou University, Kaohsiung 840, Taiwan; 6 Department of Nuclear Medicine, Kaohsiung Veterans General Hospital, Kaohsiung 813, Taiwan; hpchan@vghks.gov.tw; 7 Graduate Institute of Biomedical and Pharmaceutical Science, Fu Jen Catholic University, New Taipei City 242, Taiwan; 150110@mail.fju.edu.tw; 8 National Institute of Infectious Diseases and Vaccinology, National Health Research Institutes, Miaoli County 350, Taiwan; 9 Graduate Institute of Animal Vaccine Technology, National Pingtung University of Science and Technology, Pingtung 900, Taiwan; kpchuang@g4e.npust.edu.tw; 10 Department of Biological Science, National Sun Yat-sen University, Kaohsiung 804, Taiwan; chlee@mail.nsysu.edu.tw; 11 Mass Spectrometry Laboratory, Department of Chemistry, National University of Singapore, Singapore 119077, Singapore; chmyuch@nus.edu.sg; 12 Department of Medical Education and Research, Kaohsiung Veterans General Hospital, Kaohsiung 813, Taiwan; 13 Center of General Education, Shu-Zen Junior College of Medicine and Management, Kaohsiung 821, Taiwan; 14 Graduate Institute of Medicine, College of Medicine, Kaohsiung Medical University, Kaohsiung 807, Taiwan; 15 Institute of Medical Science and Technology, National Sun Yat-sen University, Kaohsiung 804, Taiwan; 16 Department of Medical Research, Kaohsiung Medical University Hospital, Kaohsiung 807, Taiwan; 17 Center for Cancer Research, Kaohsiung Medical University, Kaohsiung 807, Taiwan; 18 Research Center for Environmental Medicine, Kaohsiung Medical University, Kaohsiung 807, Taiwan

**Keywords:** COVID-19, asymptomatic patients, nuclear medicine, [^18^F]FDG PET/CT

## Abstract

During the coronavirus disease 2019 (COVID-19) pandemic, several case studies demonstrated that many asymptomatic patients with COVID-19 underwent fluorine-18 fluorodeoxyglucose ([^18^F]FDG) positron emission tomography/computed tomography (PET/CT) examination for various indications. However, there is a lack of literature to characterize the pattern of [^18^F]FDG PET/CT imaging on asymptomatic COVID-19 patients. Therefore, a systematic review to analyze the pulmonary findings of [^18^F]FDG PET/CT on asymptomatic COVID-19 patients was conducted. This systematic review was performed under the guidelines of PRISMA. PubMed, Medline, and Web of Science were used to search for articles for this review. Articles with the key words: “asymptomatic”, “COVID-19”, “[^18^F]FDG PET/CT”, and “nuclear medicine” were searched for from 1 January 2020 to 20 May 2021. Thirty asymptomatic patients with COVID-19 were included in the eighteen articles. These patients had a mean age of 62.25 ± 14.85 years (male: 67.71 ± 12.00; female: 56.79 ± 15.81). [^18^F]FDG-avid lung lesions were found in 93.33% (28/30) of total patients. The major lesion was [^18^F]FDG-avid multiple ground-glass opacities (GGOs) in the peripheral or subpleural region in bilateral lungs, followed by the consolidation. The intensity of [^18^F]FDG uptake in multiple GGOs was 5.605 ± 2.914 (range from 2 to 12) for maximal standardized uptake value (SUVmax). [^18^F]FDG-avid thoracic lymph nodes (LN) were observed in 40% (12/40) of the patients. They mostly appeared in both mediastinal and hilar regions with an SUVmax of 5.8 ± 2.93 (range from 2.5 to 9.6). The [^18^F]FDG uptake was observed in multiple GGOs, as well as in the mediastinal and hilar LNs. These are common patterns in PET/CT of asymptomatic patients with COVID-19.

## 1. Introduction

Coronavirus disease 2019 (COVID-19) was first detected in Wuhan, China at the end of December 2019 [1]. It is an infectious lung disease causing severe acute respiratory syndrome coronavirus 2 (SARS-CoV-2) [2,3]. Since 2019, COVID-19 has rapidly spread across the entire world. As of 23 June 2021, the World Health Organization (WHO) has estimated that there are over 178.5 million confirmed positive patients and over 3.8 million deaths [4]. Despite ongoing vaccination in many countries, more time is needed to achieve global herd immunity. A major prevention strategy for COVID-19 is still to control the transmission of SARS-CoV-2.

The common clinical symptoms of COVID-19 include fever, fatigue, dry cough, and pneumonia, as well as other rare signs such as headache, nausea, vomiting, and diarrhea [5,6]. However, some SARS-CoV-2 infected individuals are asymptomatic during COVID-19 illness [7,8]. Asymptomatic cases with COVID-19 are a source of SARS-CoV-2 transmission in social situations. They have infected more than 40% of positive cases. Since asymptomatic individuals do not present any clinical symptoms of COVID-19, it is not possible to quickly identify them for intervention. Therefore, the prevention strategy for COVID-19 is complicated due to these asymptomatic cases.

The radiopharmaceutical 2-deoxy-2-[fluorine-18] fluoro-D-glucose ([^18^F]FDG) is a glucose analog in which the normal hydroxyl group at the C-2 position in the glucose molecule is replaced with the positron-emitting radionuclide fluorine-18. Imaging is based on the detection of gamma rays after positron–electron annihilation with a positron emission tomography (PET) machine. Combined with computed tomography (CT) to assist in the localization of a lesion, PET/CT imaging with [^18^F]FDG can detect metabolic status based on degree of glucose utility in a variety of tissues. [^18^F]FDG PET/CT is commonly used for diagnosis, staging, or restaging of malignant disease due to increased glucose uptake and glycolysis in tumor cells [9,10]. Apart from malignant disease, it is also used to characterize infection and aseptic inflammation based on the high glucose uptake of activated inflammatory cells [11].

Recently, several case studies demonstrated that many asymptomatic patients with COVID-19 underwent [^18^F]FDG PET/CT examination due to other clinical indications. However, there is a lack of literature to characterize the disease pattern of [^18^F]FDG PET/CT in asymptomatic COVID-19 patients. Herein, we present a systematic review to analyze the pulmonary findings of [^18^F]FDG PET/CT in asymptomatic COVID-19 patients.

## 2. Data Sources and Review Approaches

**Figure 1:**
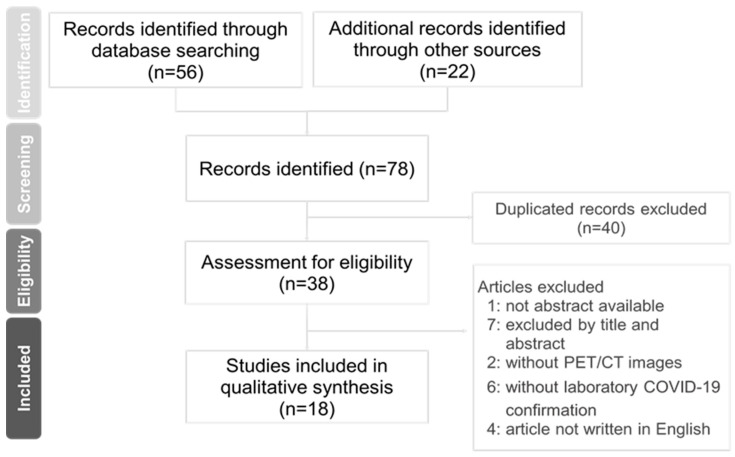
The PRISMA flow diagram of the literature selection process pertaining to this study on "asymptomatic, and FDG PET/CT " use in COVID-19 or SARS-CoV-2.

The guidelines of PRISMA were used to conduct this review (Figure 1). A literature review was performed from 1 January 2020 and up to 15 May 2021 by searching in electronic databases—mainly PubMed, Medline, and Web of Science. The central aim of this review was to detect findings of [^18^F]FDG PET/CT in asymptomatic patients with COVID-19. The types of studies in this review were restricted to case reports, case series, and retrospective case reports. The key words “asymptomatic”, “COVID-19”, “FDG PET/CT”, and “nuclear medicine” were used to search the literature for this review. Duplicated articles and articles with only abstracts were removed. Irrelevant articles were deleted after screening the titles and abstracts. All authors reviewed relevant articles and excluded those without abstract and PET/CT images, or those whose subjects did not have a laboratory-confirmed test for COVID-19. The language of all articles was limited to English.

## 3. Results

### 3.1. Selection of Studies

The PRISMA flow chart in Figure 1 summarizes the article search and selection process. We included eighteen articles from thirty-eight candidate articles. In total, 30 asymptomatic patients with COVID-19 were identified in the eighteen articles.

### 3.2. Patient Features

Table 1 characterizes the findings of [^18^F]FDG PET/CT for each patient with asymptomatic COVID-19. These patients had a mean age of 62.25 ± 14.85 years (male: 67.71 ± 12.00; female: 56.79 ± 15.81). Of the total patients, 83.33% (25/30) were enrolled from Europe, including Italy, France, Spain, Romania, and Portugal [12,13,14,15,16,17,18,19,20,21,22,23,24]; 13.33% (4/30) from North America (USA and Canada) [25,26,27,28]; and 3.33% (1/30) from Iran [29]. Of the total, 83.33% (25/30) were oncological patients who received [^18^F]FDG PET/CT under clinical indications for staging, restaging, follow-up, and for the evaluation of therapeutic response [12,13,14,15,16,18,19,20,21,22,23,24,25,27,28]. The other five patients (P9, 18, 24, 28, and 30) underwent [^18^F]FDG PET/CT for the detection of non-oncological disease or for the evaluation of clinically suspicious malignant disease [15,17,23,26,29], of which one patient (P28) was diagnosed with lung adenocarcinoma [26]. All patients were asymptomatic for COVID-19 at time of [^18^F]FDG PET/CT imaging.

### 3.3. [^18^F]FDG PET/CT Findings

Table 1 presents the findings of [^18^F]FDG PET/CT in asymptomatic patients with COVID-19. [^18^F]FDG-avid extrapulmonary lesions were found in 56.66% (17/30) of total patients. Hypermetabolic activities were separately detected in non-thoracic lymph node (LN) (n = 6: P1, 8, 14, 16, 19, and 25) [12,13,15,18,22], primary carcinoma alone (n = 5: P2, 17, 20, 22, and 29) [19,22,23,24], inflammatory tissues (n = 2: P3 and 30) [17,25], metastatic site (n = 2: P4 and 13) [12,27], primary carcinoma mixed metastatic site (n = 1: P7) [14], and primary carcinoma mixed non-thoracic lymph node (n = 1: P6) [28].

The pulmonary findings of asymptomatic patients with COVID-19 showed that increased [^18^F]FDG accumulation was detected on their lung lesion and thoracic LN. Thoracic LN with [^18^F]FDG uptake (LN involvement) was found in 40% (12/30) of total patients, excluding those with no report (15/30) and no LN involvement (2/30).

Of the total patients, 90% (27/30) demonstrated [^18^F]FDG-avid lung lesions, (except for P11 [15], 21 [20], and 24 [27].) Patients numbered P11 and P21 demonstrated different oncological diseases with no [^18^F]FDG-avid lung lesion and no above pulmonary alternation, respectively [15,22]. P24 was a non-oncological patient with unknown anosmia, and the abnormal [^18^F]FDG uptake was only detected on her right hilar LN [27]. Of these 27 patients, 11 of them had [^18^F]FDG-avid lung lesions and thoracic involvement (P6, 7, 8, 9, 10, 12, 15, 17, 18, 25, and 28). Fifteen patients with [^18^F]FDG-avid lung lesions had previously unknown performance of thoracic LN involvement (P1, 3, 4, 5, 13, 14, 16, 19, 20, 22, 23, 26, 27, 29, and 30). [^18^F]FDG-avid lung lesions alone were only detected in patient P2, with no thoracic LN involvement. Results from extrapulmonary and pulmonary findings showed that many asymptomatic COVID-19 patients with or without oncological disease had [^18^F]FDG-avid lung lesions. Some of that group also had thoracic LN involvement, mostly in patients without non-thoracic LN involvement (n = 9: P7, 9, 10, 12, 15, 17, 18, 24, and 28). A rare percentage of asymptomatic COVID-19 patients presented thoracic LN involvement alone or with no pulmonary lesions.

### 3.4. Characteristics of Pulmonary Findings 

**Figure 2:**
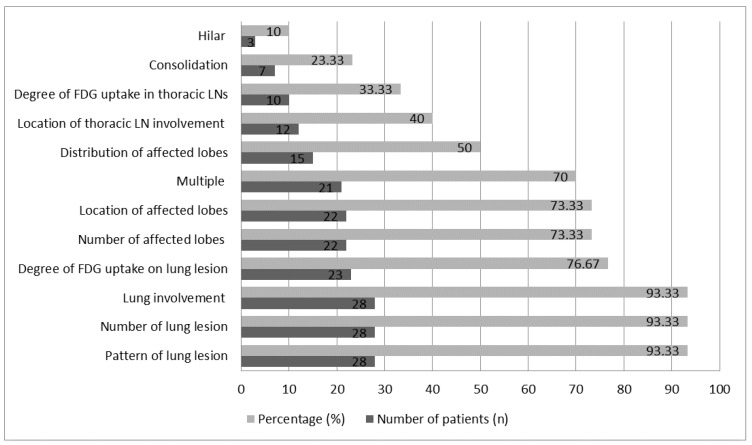
Characteristics of pulmonary findings in asymptomatic patients with COVID-19.

Figure 2 characterizes pulmonary findings in asymptomatic patients with COVID-19. Patterns of lung lesions were found in 93.33% (28/30) of total patients. The patterns of lung lesions regarding features of COVID-19 pneumonia are shown in Table 2. Ground-glass opacities (GGOs) with other lesion patterns such as consolidations, curvilinear lines, crazy paving, and lobar thickening, etc., were found on 50% (15/30) of total patients. This was slightly more than the percentage of patients with GGO alone (43.44%; 13/50). It was revealed that GGO was a major lung lesion for asymptomatic patients with COVID-19. Lung lesions were found in 93.33% (28/30) of the total patients, and 70% (21/30) of these presented with multiple lesions on their lungs, compared with 13.33% (4/30) with unique lesions and 10% (3/30) with unique lesions at multiple sites. Unique lesions were only seen in patients who had GGOs with other patterns (n = 7: P5, 7, 9, 10, 23, 26 and 30). This indicated that asymptomatic patients with COVID-19 frequently presented multiple lung lesions.

Affected lobes were recorded for 73.33% (22/30) of total patients. The percentage of patients with 1 lobe vs. 2 lobes vs. 3 lobes vs. 4 lobes vs. 5 lobes was 13.33% (1/30) vs. 23.33% (7/30) vs. 13.33% (4/30) vs. 3.33% (1/30) vs. 20% (6/30). This suggests that lesions frequently occurred on more than 3 lobes in asymptomatic patients with COVID-19. The distribution of lesions in affected lobes was studied in half of the total patients. Lesions were mostly distributed on the peripheral or subpleural region of affected lobes. In addition, the results of the location distribution of affected lobes from 73.33% (22/30) of total patients demonstrated that the infection and/or inflammation caused by COVID-19 could occur in any region in both lungs. The finding was similar to that of pulmonary involvement for asymptomatic patients with COVID-19. Of the total patients, 70% (21/30) had bilateral lung involvement. These findings indicated that lung lesions were detected on any lobe, either in the peripheral or subpleural regions of lung, in asymptomatic patients with COVID-19.

In this systematic review, most COVID-19 patients were incidentally detected by [^18^F]FDG PET/CT scan, which was originally used for tumor staging or determination of suspicious disease progression. Differentiation between lung metastasis and COVID-19 infection is important but difficult to achieve for physicians, especially for patients who underwent [^18^F]FDG PET/CT. The number of affected lung nodules may be single or multiple in metastasis and/or COVID-19 patients. However, metastatic lung nodules may appear round-shaped, well-circumscribed, with variability in size and soft tissue attenuation, mainly in the peripheral region of the lung. In our observations, 70% of COVID-19 patients presented multiple GGOs involving bilateral lung abnormalities with corresponding mild-to-hot [^18^F]FDG uptake. This indicated that COVID-19 infection was related to glucose hypermetabolism. The location of GGOs could be peripherally distributed with lower lobe involvement. Other patterns were observed in COVID-19 patient’s lungs, including lung consolidation, linear opacity, septal thickening, tree-in-bud opacity, or pleural thickening; all of these showed mild-to-moderate [^18^F]FDG uptake. It is different in main lung involvement; cavitation is not frequently noted in these patients, dissimilarly to patients with tuberculosis infection or squamous cell carcinoma. Nevertheless, its appearance suggests lung injury. We suggest suspecting individuals with characteristics of [^18^F]FDG uptake appearance in lung and specific CT scan morphology of being COVID-19 patients. Accordingly, these patients should proceed to screening for SARS-CoV-2 infection.

Table 2 shows the features of LN involvement, which randomly occurred as diverse patterns in lung lesions. The location of thoracic LN involvement was frequently in both sides of the mediastinal and hilar regions with or without other thoracic LN, i.e., subclavian or carinal LNs (Table 2), except for the above characterization of [^18^F]FDG-avid lung lesions or thoracic LN. The intensity of [^18^F]FDG uptake was separately analyzed on lung lesions and thoracic LNs. Maximal standardized uptake value (SUVmax), maximal SUV based on body weight (SUV bw max), or maximal SUV based on lean body mass (SUV lbm max) were used to represent the degree of [^18^F]FDG uptake in the lung lesion or thoracic LNs. The range of [^18^F]FDG uptake in lung lesions was from 2 to 12 for SUVmax, 3.3 to 10.7 for SUV bw max, and 3.7 to 6.8 for SUV lbm max, respectively. The SUVmax in thoracic LNs ranged from 2.5 to 9.6, while that of SUV bw max ranged from 3.9 to 5.9. These findings demonstrate that asymptomatic patients with COVID-19 presented various degrees of [^18^F]FDG uptake in both lung lesions and regions of involved thoracic LNs.

### 3.5. Patient Management and Hospital Infection Control

Table 3 summarizes the patient management for asymptomatic patients with COVID-19 detected by [^18^F]FDG PET/CT and hospital infection control. Asymptomatic patients were from 17 hospitals in six countries. For patient management, most hospitals in these countries required immediate laboratory confirmation of COVID-19 infection by RT- PCR, PCR, or even additional verification by serological test, and then asymptomatic patients were home quarantined [12,13,14,15,19,20,22,23,24,25,26,27,28]. However, some hospitals did not emphasize the status of being under quarantine [12,13,19,22,24,25,26]. Of these cases, P27 was home quarantined before the laboratory confirmation of COVID-19 infection [20]. Subsequently, P27 was confirmed later due to symptom onset. In addition, some hospitals in Europe referred the patients to a dedicated COVID-19 medical facility for laboratory COVID-19 confirmation [13,15,22] and COVID-19 treatment [13,22]. Patients at registered hospitals were also provided with COVID-19 treatment [12,18,20]. Oncological therapy was postponed for some patients with COVID-19 [12,22].

As the radiologists had insufficient experience in COVID-19 imaging on [^18^F]FDG PET/CT, two patients (P5 and P6) from hospitals in Italy were not immediately confirmed by laboratory COVID-19 testing [16,21]. This also happened to P30 at another hospital in Spain [17]. P6 and P30 were confirmed to have SARS-CoV-2 infection later due to symptom onset. However, this was over four days after the [^18^F]FDG PET/CT primary imaging. Although most hospitals immediately confirmed asymptomatic patients with COVID-19 infection after [^18^F]FDG PET/CT scan, this does not rule out the fact that radiologists, due to insufficient experience in COVID-19 imaging on [^18^F]FDG PET/CT, may delay diagnosis for asymptomatic patients by [^18^F]FDG PET/CT and thus increase the risk of the spread of COVID-19.

Three hospitals were developing guidelines for hospital infection control since encountering asymptomatic patients with COVID-19 through [^18^F]FDG PET/CT examination [14,19,28]. These three hospitals were in Portugal [14], France [19], and the USA [28]. The guidelines for hospital infection control are presented in Table 3. Two hospitals screened for the COVID-19 infection risk of every patient before and upon entering the facility [14,19]. All hospitals canceled radiopharmaceutical imaging investigations and cleaned the imaging room after having suspected or confirmed cases of COVID-19 [14,19,28]. The hospital in Portugal even cleaned the imaging equipment after each use [14]. In addition, employees were required to self-monitor after having contact with suspected cases of COVID-19 [19,28]. These guidelines expanded the prevention targets to each patient and also increased the disinfection level. Overall, it is suggested to take extensive measures to avoid the spread of COVID-19 via contact with asymptomatic patients.

## 4. Discussion

This systematic review aimed to analyze the pulmonary findings of [^18^F]FDG PET/CT in asymptomatic patients with COVID-19. We included 30 asymptomatic patients with COVID-19 from eighteen articles. The patterns of [^18^F]FDG PET/CT appearance in COVID-19 pneumonia were similar to those of asymptomatic patients with or without oncological diseases. There were two common pulmonary patterns of [^18^F]FDG PET/CT in asymptomatic COVID-19 patients. One pattern was the wide range of [^18^F]FDG uptake on both peripheral and subpleural lungs, corresponding to multiple GGOs. Another feature was [^18^F]FDG-avid thoracic LNs with various degrees of glucose metabolism, in which [^18^F]FDG uptake frequently occurred in both sides of mediastinal and hilar LNs.

GGOs on both peripheral and subpleural lungs was the most common feature of chest CT on asymptomatic COVID-19 patients [30] and is consistent with our findings on PET/CT. Besides in asymptomatic COVID-19 patients, GGOs are also found in other infectious diseases with a variable degree of [^18^F]FDG uptake [31]. Abnormal [^18^F]FDG uptake in mediastinal and hilar LNs is found in malignant diseases such as lung cancer and lymphoma [32,33], and in acute primary pulmonary histoplasmosis [34]. LN alternation on chest CT is commonly found in patients with severe COVID-19 [35]. It was first found on [^18^F]FDG PET/CT for asymptomatic patients with COVID-19.

Some limitations in this review should be considered. First, during the time of this review’s preparation, new and updated findings were released which are not included. Second, most asymptomatic cases with COVID-19 were from Europe, and cases from other areas should also be investigated.

## 5. Conclusions

This review demonstrates that asymptomatic COVID-19 patients present a wide range of [^18^F]FDG uptake patterns in multiple GGOs and on both sides of mediastinal and hilar LNs. Although these patterns can be also found in other infectious or malignant diseases, the [^18^F]FDG PET/CT discloses the increased metabolic status that is revealed in asymptomatic COVID-19 patients. Nuclear medicine staff should consider the risk of the spread of COVID-19 during [^18^F]FDG PET/CT examination. Every patient should be considered possibly infectious and treated with universal precautions. This study emphasizes the need for such universal precautions.

## Figures and Tables

**Table 1:** Characteristics of [^18^F]FDG PET/CT studies in asymptomatic patients with COVID-19.

Patients No./Sex/ Age (y)	Reference	Country	Clinical Disease	[^18^F]FDG PET/CT Indications	Extrapulmonary PET/CT Findings	The Pattern of PET/CT Finding in Pulmonary Regions
**P1/M/70**	Boulvard Chollet XLE et al., 2021 [13]	Italy	Hodgkin Lymphoma	Staging	[^18^F]FDG-avid uptake in bilateral cervical lymphadenopathy, predominantly in the left side (SUVmax 9.0)	Tree-in-bud opacities and peripheral and subpleural GGOs in both lungs, corresponding to mild [^18^F]FDG uptake (SUVmax 2.4)
**P2/F/80**	Habouzit V et al., 2020 [19]	France	Anal canal adenocarcinoma	Staging	Prominent [^18^F]FDG-avid tumor uptake in anal canal (SUVmax 12.8)	Subpleural patchy, rounded, and diffuse GGOs in right lung, with mildly diffuse [^18^F]FDG uptake (SUV max 2.4)
**P3/F/69**	Pillenahalli Maheshwar- appa R et al., 2021 [25]	USA	Multiple myeloma	Follow-up	Uneven hot [^18^F]FDG uptake in right maxillary sinus, favored sinusitis	Prominent [^18^F]FDG-avid uptake of extensive GGOs in peripheral to subpleural region over bilateral lower lobes (SUVmax 12.0)
**P4/M/87**	Krebs S et al., 2020 [27]	USA	Primary salivary duct carcinoma	Restaging	Prominent [^18^F]FDG-avid uptake in hepatic and colon metastases	Prominent [^18^F]FDG-avid uptake in multiple GGOs and patchy opacities, with intralobular septal thickening of lung in LUL and RLL
**P5/M/59**	Mattoli MV et al., 2020 [21]	Italy	Diffuse B cell lymphoma	Therapeutic response	NR	Focally increasing [^18^F]FDG uptake with a consolidation surrounding a faint GGO in central RML (SUVmax 3.3)
**P6/M/67**	Johnson LN et al., 2020 [28]	USA	Hereditary nonpolyposis colorectal cancer	Follow-up	Mild [^18^F]FDG uptake in the long segment of the small bowel at the mid-abdomen (SUVmax 4.7) and faint [^18^F]FDG uptake in lymph nodes at the lower abdomen (SUVmax 2.9)	Uneven [^18^F]FDG-avid uptake of GGOs in both lungs (SUVmax 9.5), mediastinal and hilar LNs (SUVmax 9.6)
**P7/F/65**	Castanheira J et al., 2020 [14]	Portugal	Breast cancer	Follow-up	Recurrent breast cancer and bone marrow metastasis	Hot [^18^F]FDG uptake of a GGO in the RLL with interlobular thickening (SUVmax 8.0) and moderate increasing [^18^F]FDG uptake in ipsilateral hilar and subcarinal LN (SUVmax 4.0 to 5.0)
**P8/M/54**	Colandrea M et al., 2020 [15]	Italy	Non-hodgkin lymphoma	Staging	Pathological increasing [^18^F]FDG uptake in right inguinal lymphadenopathy	Moderate-to-hot [^18^F]FDG uptake of GGOs in lower lobes of both lungs (SUV bw max 10.7); faint [^18^F]FDG uptake of focal consolidation in LUL (SUV bw max 3.9). Mediastinum and left subclavian LNs (SUV bw max 4.6)
**P9/M/61**	Colandrea M et al., 2020 [15]	Italy	-	Characterization of unknow brain and lung LN lesion	NR	Mild-to-moderate [^18^F]FDG uptake including consolidation in RUL (SUV bw max 3.6), multiple GGOs in LLL (SUV bw max 4.9), mediastinal, carinal, and hilar LNs (SUV bw max 3.9)
**P10/M/48**	Colandrea M et al., 2020 [15]	Italy	Lung cancer	Restating	Increased hot [^18^F]FDG uptake in retro-bronchial LNs (SUV bw max 9.8), favored recent radiotherapy related	Mild-to-moderate [^18^F]FDG uptake including consolidation in LUL (SUV bw max 3.3), multiple peripheral GGOs and septal thickening in LLL (SUV bw max 6.5) Mediastinal and left hilar LNs (SUV bw max 5.2)
**P11/M/54**	Colandrea M et al., 2020 [15]	Italy	Melanoma	Follow-up	NR	Multiple small GGOs without corresponding [^18^F]FDG uptake in both lungs
**P12/NR/NR**	Colandrea M et al., 2020 [15]	Italy	Tongue cancer	Follow-up	NR	Increased [^18^F]FDG uptake in peripheral and parenchymal GGOs of both lower lobes (SUV bw max 6.3), focal consolidation in RUL (SUV bw max 6.8) and right hilar LNs (SUV bw max 5.9)
**P13/F/56**	Albano D et al., 2020 [12]	Italy	Anal cancer	restaging	An inguinal metastatic nodule with corresponding [^18^F]FDG uptake	Mild [^18^F]FDG uptake of GGOs and consolidation in both lower lobes (SUVmax 3.6)
**P14/M/77**	Albano D et al., 2020 [12]	Italy	Laryngeal cancer	Staging	Increased hot [^18^F]FDG uptake in right epiglottis and local cervical nodes	Faint [^18^F]FDG uptake of GGOs in both lungs (SUVmax 2.0)
**P15/F/65**	Albano D et al., 2020 [12]	Italy	Ovarian cancer	Follow-up	No abnormal [^18^F]FDG uptake in primary site of ovarian malignancy	[^18^F]FDG-avid uptake of corresponding GGOs in both lungs (SUVmax 6.9)
**P16/F/55**	Albano D et al., 2020 [12]	Italy	Hodgkin lymphoma	Therapeutic response	Increasing [^18^F]FDG uptake in the axillary nodes, suggestive of lymphoma nodule	[^18^F]FDG-avid uptake of corresponding GGOs in right lung (SUVmax 5.0)
**P17/F/57**	Scarlattei M et al., 2020 [23]	Italy	Breast cancer	Restaging	[^18^F]FDG-avid tumor uptake in left breast (SUVmax 6.5)	Multiple mild-to-hot [^18^F]FDG uptake of GGOs in bilateral lower lobes (range of SUVmax 2.2 to 9.1), and moderate-to-hot [^18^F]FDG uptake in mediastinal, hilar, and carinal LNs (SUVmax 7.0)
**P18/F/57**	Scarlattei M et al., 2020 [23]	Italy	-	Characterization of splenic lesion	No abnormal [^18^F]FDG uptake in splenic lesion	Several GGOs in both lungs with mild [^18^F]FDG uptake (SUVmax 4.6) and faint [^18^F]FDG uptake in bibasilar LNs (SUVmax 2.5)
**P19/F/38**	Piciu A et al., 2021 [22]	Romania	Nasopharyngeal carcinoma	Therapeutic response	[^18^F]FDG-avid uptake in left supraclavicular lymphadenopathy	Faint [^18^F]FDG uptake of multiple GGOs in RML
**P20/M/70**	Piciu A et al., 2021 [22]	Romania	Maxillary sinus carcinoma	Restaging	[^18^F]FDG-avid nodules in maxillary sinus	Hot [^18^F]FDG uptake of multiple GGOs in both lungs (SUV lbm max 6.8)
**P21/F/64**	Piciu A et al., 2021 [22]	Romania	Follicular thyroid carcinoma	Follow-up	NR	A metastasis-associated nodule with minimally increased [^18^F]FDG uptake in peripheral RLL and without other lung alterations
**P22/F/65**	Piciu A et al., 2021 [22]	Romania	Rectal cancer	Staging	[^18^F]FDG-avid uptake in rectal area, favored rectal malignant	Diffuse mild GGOs in both lungs (SUV lbm max 3.8)
**P23/F/70**	Piciu A et al., 2021 [22]	Romania	Breast cancer	Follow-up	NR	Diffuse GGOs with crazy paving in both lungs with mild [^18^F]FDG uptake (SUV lbm max 3.7)
**P24/F/27**	Karimi-Galougahi M et al., 2020 [29]	Iran	-	Characterization of unknow anosmia	NR	Right hilar LN (SUVmax 2.6) with mild [^18^F]FDG uptake, suspicious occult malignancy, then confirmation of a transient [^18^F]FDG-avid LN by scan 10 days later
**P25/M/88**	Ferrando-Castagnetto F et al, 2020 [18]	Spain	Melanoma	Follow-up	Intense [^18^F]FDG-avid uptake in both left preauricular and bilateral cervical lymphadenopathy No changes in melanoma associated features	Mild [^18^F]FDG uptake of GGOs in both lungs and curvilinear lines in right lung (SUVmax 3.2); hot [^18^F]FDG uptake in mediastinal and bilateral hilar lymphadenopathy (largest size 9 mm, SUVmax 8.1)
**P26/NR/NR**	Cosma L et al., 2020 [16]	Italy	Colorectal cancer	Therapeutic response	NR	Multiple GGOs in right lung and a nodule in right interlobar region, which showed mild [^18^F]FDG uptake (SUVmax 2.4)
**P27/M/68**	López-Mora DA et al., 2021 [20]	Spain	Urothelial carcinoma	Follow-up	No abnormal [^18^F]FDG uptake outside thoracic cavity	Multiple mild [^18^F]FDG uptake of GGOs in both lungs, then disappeared 3 months later
**P28/M/67**	Martineau P, et al., 2020 [26]	Canada	-	Characterization of a suspicious lung nodule	No abnormal [^18^F]FDG uptake outside thoracic cavity	Focal increased [^18^F]FDG uptake of lung nodule (SUVmax 4.1), confirming adenocarcinoma by biopsy; moderate-to-hot [^18^F]FDG uptake of GGOs and consolidative opacities in both lungs (SUVmax from 5.0 to 7.2), also in mediastinal LNs, more likely reactive LNs by size evaluation
**P29/F/27**	Simand C et al., 2020 [24]	France	Hodgkin lymphoma	Restaging	Increased [^18^F]FDG uptake in bone marrow	GGOs with consolidations in LLL (SUVmax 8.1)
**P30/M/78**	de Barry O et al., 2020 [17]	France	-	Characterization of rheumatic polymyalgia	Increased [^18^F]FDG uptake of osteoarticular involvement in both shoulders	A GGO with consolidation in LUL with focal hot [^18^F]FDG uptake (SUVmax 5.4)

M, male; F, female; PET/CT, positron emission tomography–computed tomography; CT, computed tomography; -, without_,_; GGO, ground-glass opacity; RUL, right upper lobe; RML, right middle lobe; RLL, right lower lobe; LUL, left upper lobe; LLL, left lower lobe; LN, lymph node; NR, not reported; SUVmax, maximal standardized uptake values; SUV bw max, maximal standardized uptake values in body weight; SUV lbm max, maximal standardized uptake values in lean body mass.

**Table 2:** Characteristics of pulmonary patterns with [^18^F]FDG PET/CT in asymptomatic patients with COVID-19.

Pattern of Lung Lesion	Number of Lung Lesions	Number of Affected Lobes	Distribution of Affected Lobes	Location of Affected Lobes	Lung Involvement	Location of Thoracic LN Involvement	Degree of [^18^F]FDG Uptake	References and Images
**Without lung lesion (n = 2)** **(P21, P24)**	-	-	-	-	-	Hilar alone (n = 1) (P24) NR (n = 1)	LN: SUVmax 2.6 (n = 1) (P24) NR (n = 1)	[22]: Figure 4 (P21) [29]: Figure 1 (P24)
**GGO alone (n = 13)** **(P2, P3, P6, P11, P14-20, P22, P27)**	Multiple (n = 13)	1 lobe (n = 2) (P2, P19) 2 lobes (n = 2) (P3, P17) 3 lobes (n = 1) (P4) 5 lobes (n = 4) (P11, P15, P20, P22) NR (n = 4)	PS (n = 1) (P6) SS (n = 4) (P2, P16-18) PS and SS (n = 1) (P3) NR (n = 7)	RML (n = 1) (P19) RLL (n = 2) (P2, P6) RLL and LLL (n = 2) (P3, P17) Right lung lobes (n = 1) (P16) All lung lobes (n = 4) (P11, P15, P20, P22) NR (n = 3)	Unilateral (n = 3) (P2, P16, P19) Bilateral (n = 10) (P3, P6, P11, P14, P15, P17 P18, P20, P22, P27)	No involvement (n = 2) (P2, P11) Hilar alone (n = 1) (P18) Mediastinal alone (n = 1) (P15) Mediastinal and hilar (n = 1) (P6) Mediastinal, hilar, and carinal (n = 1) (P17) NR (n = 7)	Lung lesion: No [^18^F]FDG uptake (n = 1) (P11) SUV max 2 to 12 (n = 8) (P2, P3, P6, P14-18) SUV lbm max 6.8 (n = 1) (P20) NR (n = 3) (P19, P20, P27) LN: SUVmax 2.5 to 9.6 (n = 3) (P6, P17, P18) NR (n = 10)	[12]: Figures 2, 4 and 6 (P14, P15, P16) [15]: Figure 3 (P11) [19,20,25,28]: Figure 1 (P2, P3, P6, P27) [22]: Figures 2, 3 and 5 (P19, P20, P22) [23]: Figure 2 and Supplementary Figure 3 (P17, P18)
**GGO plus consolidation (n = 7)** **(P5, P8, P9, P12, P13, P28, P29)**	Unique (n = 1) (P5) Multiple (n = 5) (P8, P12, P13, P28, P29) Mixed (n = 1) (P9)	1 lobe (n = 1) (P5) 2 lobes (n = 3) (P9, P13) 3 lobes (n = 2) (P8, P12) 5 lobes (n = 1) (P28)	PS (n = 3) (P8, P12, P28) SS (n = 1) (P29) NR (n = 3) (P5, P9, P13)	RML (n = 1) (P5) RUL and LLL (n = 2) (P9, P29) RLL and LLL (n = 1) (P13) RUL, RLL, and LLL (n = 1) (P12) RLL and left lung lobes (n = 1) (P8) All lung lobes (n = 1) (P28)	Unilateral (n = 1) (P5) Bilateral (n = 6) (P8, P9, P12, P13, P28, P29)	Hilar alone (n = 1) (P12) Mediastinal and left subclavian (n = 1) (P8) Mediastinal, hilar, and carinal (n = 1) (P9) Mediastinal lymphadenopathy (n = 1) (P28) NR (n = 3)	Lung lesion: SUVmax 3.3 to 8.1 (n = 4) (P5, P13, P28, P29) SUV bw max 3.6 to 10.7 (n = 3) (P8, P9, 12) LN: SUV bw max 3.9 to 5.9 (n = 3) (P8, P9, P12) NR (n = 4)	[12,21,26]: Figure 1 (P5, P13, P28) [15]: Figures 1 and 4 (P8, P9, P12) [24]: Figures 1 and 2 (P29)
**GGO plus consolidation and** **septal thickening (n = 1) (P10)**	Mixed	2 lobes	PS	LUL and LLL	Unilateral	Mediastinal and hilar	Lung lesion: SUV bw max 3.3 (consolidation); 6.5 (GGOs) LN: SUV bw max 5.2	[15]: Figure 2
**GGO plus consolidation and** **curvilinear line (n = 1) (P30)**	Unique	4 lobes	NR	Right lung lobes and LUL	Bilateral	NR	Lung lesion: SUVmax 5.4	[17]: Figure 1
**GGO plus interlobular** **thickening (n = 1) (P7)**	Unique	1 lobe	PS	RLL	Unilateral	Ipsilateral hilar and subcarinal	Lung lesion: SUVmax 8 LN: SUVmax 5	[14]: Figure 1
**GGO plus intralobular thickening (n = 1) (P4)**	Multiple	2 lobes	NR	RLL and LUL	Bilateral	NR	NR	[27]: Figure 1
**GGO plus tree-in-bud opacities** **(n = 1) (P1)**	Multiple	NR	PS and SS	NR	Bilateral	NR	Lung lesion: SUVmax 2.4	[13]: Figure 1
**GGO plus crazy paving (n = 1) (P23)**	Unique	5 lobes	NR	All lung lobes	Bilateral	NR	Lung lesion: SUV lbm max 3.7	[22]: Figure 6
**GGO plus a nodule (n = 1) (P26)**	Mixed	3 lobes	PS	Right lung lobes	Unilateral	NR	Lung lesion: SUVmax 2.4 (GGOs)	[16]: Figures 1 and 2
**GGO plus curvilinear line,** **thrombus, and lung** **infiltrates (n = 1) (P25)**	Multiple	NR	SS and CS	NR	Bilateral	Mediastinal and hilar	Lung lesion: SUVmax 3.2 LN: SUVmax 8.1 (mediastinal)	[18]: Figure 1

-, without; PS, peripheral site; SS, subpleural site; CS, central site; NR, not reported.

**Table 3:** Patient management for asymptomatic patients with COVID-19 detected by [^18^F]FDG PET/CT and hospital infection control.

Reference	Facility	Country	Patients No. (Reference Table 1)	Patient Management	Hospital Infection Control
**Boulvard Chollet XLE et al., 2021 [13]**	University Hospital San Pedro and Centre	Italy	P1	Immediately isolated, RT-PCR testing, and started COVID-19 treatment with paracetamol and hydroxychloroquine sulphate (dolquine), plus omeprazole, enoxaparin, furosemide, azithromycin, and tranxilium	NR
**Mattoli MV et al., 2020 [21]**	NR	Italy	P5	Chest X-ray for reevaluation, then RT-PCR testing immediately and home quarantine	NR
**Colandrea M et al., 2020 [15]**	European Institute of Oncology IRCCS	Italy	P8, 9, 10, 11, and 12	Laboratory COVID-19 testing immediately in dedicated COVID-19 medical units (P8 and P9), another center (P10), or hospitals (P11 and P12), and home quarantine for all patients	NR
**Albano D et al., 2020 [12]**	Spedali Civili Brescia	Italy	P13, 14, 15, and 16	All patients: RT-PCR testing immediately P13 and P14: postponed oncological therapy, referred to dedicated COVID-19 medical unit, and started COVID-19 treatment with hydroxychloroquine plus ritonavir-lopinavir	NR
**Scarlattei M et al., 2020 [23]**	University Hospital of Parma	Italy	P17 and P18	Immediate home quarantine, and retrospectively confirmed by serology test	NR
**Cosma L et al., 2020 [16]**	NR	Italy	P26	RT-PCR was performed at the time of symptom development	NR
**Habouzit V et al., 2020 [19]**	CHU Saint-Etienne	France	P2	RT-PCR testing immediately	Screened the COVID-19 infection risk for every patient before and upon entering the unit, cleaned the imaging equipment after every using, cancel nonurgent investigations when pervious patients with confirmed or suspected COVID-19, notified the suspected COVID-19 and their contacted person should be self-monitoring
**Simand C et al., 2020 [24]**	University Hospital of Strasbourg	France	P29	FDG PET/CT scanning again at ten days after the time of first imaging	NR
**de Barry O et al., 2020 [17]**	Ambroise ParéTeaching Hospital	France	P30	PCR test was performed at time of symptoms developed	NR
**Piciu A et al., 2021 [22]**	NR	Romania	P19, 20, 21, 22, and 23	PCR testing immediately, postponed oncological therapy, referred to dedicated COVID-19 medical units and started the COVID-19 treatment with azithromycin plus chloroquine	NR
**Ferrando-Castagnetto F et al, 2020 [18]**	Hospital de Clínicas Dr. Manuel Quintela	Spain	P25	RT-PCR testing immediately and started COVID-19 treatment with ceftriaxone, azithromycin, and methylprednisolone for five days in hospital, then prolonged subcutaneous treatment with enoxaparin at home	NR
**López-Mora DA et al., 2021 [20]**	Hospital de la Santa Creu i Sant Pau	Spain	P27	Home quarantine, RT-PCR was performed at the time of symptom development, and received incomplete COVID-19 treatment with hydroxychloroquine and oxygen-therapy due to voluntary abandonment	NR
**Castanheira J et al., 2020 [14]**	Champalimaud Centre for the Unknown	Portugal	P7	RT-PCR testing immediately and home quarantine	Separated the employees into the rotational teams, screened the COVID-19 infection risk for every patient before and upon entering the facility, canceled the radiopharmaceutical imaging investigation when previous patients were confirmed or suspected to have COVID-19
**Karimi-Galougahi M et al., 2020 [29]**	NR	Iran	P24	RT-PCR testing immediately	NR
**Martineau P, et al., 2020 [26]**	Health Sciences Centre Winnipeg	Canada	P28	PCR testing immediately	NR
**Pillenahalli Maheshwar-** **appa R et al., 2021 [25]**	University of Iowa Hospitals and Clinics	USA	P3	RT-PCR testing immediately and home quarantine	NR
**Krebs S et al., 2020 [27]**	Memorial Sloan Kettering Cancer Center	USA	P4	RT-PCR testing immediately and home quarantine	NR
**Johnson LN et al., 2020 [28]**	NR	USA	P6	RT-PCR testing immediately and home quarantine	If it had received patients with confirmed or suspected COVID-19, the imaging room would be closed for one hour and cleaned by a high-efficiency particulate air (HEPA filter). Employees exposed to COVID-19 were required to self-screen daily and return to work when they were asymptomatic or had a lack of relevant symptoms

NR, not reported.

## Data Availability

The data presented in this study are available on request from the corresponding author.
